# pTDP‐43 aggregates accumulate in non‐central nervous system tissues prior to symptom onset in amyotrophic lateral sclerosis: a case series linking archival surgical biopsies with clinical phenotypic data

**DOI:** 10.1002/cjp2.297

**Published:** 2022-10-13

**Authors:** Samuel B Pattle, Judi O'Shaughnessy, Owen Kantelberg, Olivia M Rifai, Judith Pate, Kristine Nellany, Nadine Hays, Mark J Arends, Mathew H Horrocks, Fergal M Waldron, Jenna M Gregory

**Affiliations:** ^1^ NHS Lothian Department of Pathology Edinburgh UK; ^2^ Centre for Clinical Brain Sciences University of Edinburgh Edinburgh UK; ^3^ EaStCHEM School of Chemistry University of Edinburgh Edinburgh UK; ^4^ Translational Neuroscience PhD Program, Centre for Clinical Brain Sciences University of Edinburgh Edinburgh UK; ^5^ Centre for Discovery Brain Sciences University of Edinburgh Edinburgh UK; ^6^ Department of Pathology NHS Grampian Aberdeen UK; ^7^ NHS Grampian Biorepository Aberdeen UK; ^8^ Edinburgh Pathology, Institute of Genetics & Cancer University of Edinburgh Edinburgh UK; ^9^ Institute of Medical Sciences University of Aberdeen Aberdeen UK

**Keywords:** ALS, TDP‐43, gastrointestinal tract, non‐CNS tissue, human tissue, pathology

## Abstract

Neurodegenerative diseases such as Parkinson's disease (PD), Alzheimer's disease (AD), and amyotrophic lateral sclerosis (ALS) are traditionally considered strictly neurological disorders. However, clinical presentation is not restricted to neurological systems, and non‐central nervous system (CNS) manifestations, particularly gastrointestinal (GI) symptoms, are common. Our objective was to understand the systemic distribution of pathology in archived non‐CNS tissues, taken as part of routine clinical practice during life from people with ALS. We examined tissue from 13 people who went on to develop ALS; including sporadic ALS (*n* = 12) and *C9orf72* hexanucleotide repeat expansion (*n* = 1). The tissue cohort consisted of 68 formalin‐fixed paraffin embedded samples from 21 surgical cases (some patients having more than one case over their lifetimes), from 8 organ systems, which we examined for evidence of phosphorylated TDP‐43 (pTDP‐43) pathology. We identified pTDP‐43 aggregates in multiple cell types of the GI tract, including macrophages and dendritic cells within the lamina propria; as well as ganglion/neuronal and glial cells of the myenteric plexus. Aggregates were also noted within lymph node parenchyma, blood vessel endothelial cells, and chondrocytes. We note that in all cases with non‐CNS pTDP‐43 pathology, aggregates were present prior to ALS diagnosis and in some instances preceded neurological symptom onset by more than 10 years. These data imply that patients with microscopically unexplained non‐CNS symptoms could have occult protein aggregation that could be detected many years prior to neurological involvement.

## Introduction

Neurodegenerative diseases such as Parkinson's disease (PD), Alzheimer's disease (AD), and amyotrophic lateral sclerosis (ALS) are traditionally classified and managed as neurological disorders. There is, however, marked clinical heterogeneity within these disorders, which may in part contribute to the large number of failed clinical trials, and clinical presentation is not restricted to neurological dysfunction [1]. Indeed, non‐central nervous system (CNS) manifestations, including gastrointestinal (GI) symptoms (e.g. weight loss and altered bowel habit), are common in these patients [1]. Importantly, to date, biomarker and therapeutic studies in the neurodegeneration field have been primarily aimed at assessing pathways known to be perturbed within the CNS, and in a clinically diverse patient population. It should be possible to broaden the therapeutic potential for people with neurodegenerative diseases by expanding our approach to include clinical trials targeted at non‐motor symptoms, with the aim of improving quality of life and even reducing the non‐CNS symptom burden for people with ALS. This approach demands a deeper understanding of the systemic distribution and molecular drivers of these non‐CNS symptoms.

Systemic non‐CNS manifestations of PD and other neurodegenerative diseases are now well established [2, 3]. Furthermore, a population‐wide study of almost 1 million people in a Swedish cohort between 1965 and 2016 demonstrated that individuals with GI symptoms who underwent a colonoscopy with biopsies that were reported as histologically normal were statistically at a higher risk of developing ALS (hazard ratio = 1.22; 95% confidence interval = 0.94–1.51) [4]. These data imply that patients with GI symptoms could have occult protein aggregation that could be detected diagnostically many years prior to neurological involvement. Indeed, alpha‐synuclein has been shown to accumulate in pathological aggregates in the GI tract of PD patients up to 20 years before CNS manifestations begin [5], raising the possibility that such biomarkers may also hold promise as early indicators of disease.

The unifying proteinopathy seen in all cases of sporadic ALS (sALS) and most genetic ALS cases is the cytoplasmic accumulation of phosphorylated TDP‐43 (pTDP‐43) aggregates in the CNS [6]. Several studies have demonstrated pTDP‐43 aggregates within muscle and peripheral nerve biopsies from patients with ALS, using western blotting, immunohistochemistry, and electron microscopy [7, 8, 9]. Furthermore, several TDP‐43 animal models have demonstrated GI pathology that occurs prior to neurological symptom onset [10, 11, 12]. However, no study to date has evaluated human GI tissue or other non‐CNS tissues in a systematic way in this patient group. Here, we examine non‐CNS tissues, including GI specimens, from a cohort of people with ALS to assess the systemic distribution and burden of TDP‐43 pathology in archived tissues taken as part of routine clinical practice during life from people with ALS.

## Methods

### Ethics

All tissue was requested from the Lothian NRS BioResource RTB (ethical approval 15/ES/0094) and the Grampian Biorepository (ethical approval 21/NS/0047), covering use of residual tissue surplus to diagnostic requirements taken as standard of care with approval number SR1684 and TR000301. All archived formalin‐fixed, paraffin‐embedded tissue material with an NHS Lothian/NHS Grampian diagnostic code (e.g. any tissue samples taken for diagnostic or surgical purposes at any time during the patient's life, under the care of NHS Lothian and NHS Fife) was requested for 48 patients with known diagnosis of sALS or ALS with a *C9orf72* hexanucleotide repeat expansion, and three age and sex matched non‐ALS colon biopsies. The tissues were assessed for sufficiency by two independent histopathologists (SBP and JMG) using H&E stained sections. All clinical data, in addition to data provided by the Lothian NRS BioResource, were non‐identifiable and collected as part of Scottish Motor Neuron Disease Register and Care Audit Research and Evaluation for Motor Neuron Disease platform (ethics approval from Scotland A Research Ethics Committee 10/MRE00/78 and 15/SS/0216), and all patients consented to the use of their data during life. All anonymised demographic and clinical data are collated in Table 1. Tissue provided by the Biorepositories had been fixed in 10% formalin for a minimum of 72 h, then dehydrated in an ascending alcohol series (70–100%) followed by three 4‐h washes in xylene. Three successive 5‐h paraffin wax embedding stages were performed followed by cooling and sectioning of tissue on a Leica microtome into 4 × 4 μm thick serial sections that were collected on Superfrost microscope slides (ThermoFisher Scientific, Perth, UK). Sections were dried overnight at 40 °C before immunostaining.

**Table 1:** Demographic and pathological data for the 13 ALS patients comprising the ante‐mortem tissue cohort examined

Patient ID	Cohort	Number of tissue blocks	Age at sampling	Age at death	Specimen type	Diagnosis	pTDP‐43 pathology	Cell types/regions affected	If TDP‐43 present – time to:	
									Symptom onset	ALS diagnosis
1	sALS	2	62	65	Colon	Normal	Present	Myenteric plexus and lamina propria	Coincident	2 months
		6	62		Muscle	Denervation	Present			
3	sALS	6	53	72	Thyroid	Goitre (adenomatous)	Absent	Endothelial cells		
11	sALS	1	45	59	Endometrium	Endometrium (secretory)	Absent			
		1	57		Endometrium	Endometrium (inactive)	Absent			
14	sALS	1	54	61	Skin	Seborrheic keratosis	Present	Dendritic cells; endothelial cells; nerve bundles	48 months	60 months
17	sALS	1	44	56	Cervix	Polyp	Absent			
		1	44		Skin	Seborrheic keratosis	Absent			
		6	46		Uterus	Leiomyoma	Absent			
18	SALS	2	73	74	Urethra	Chronic inflammation	Absent			
26	sALS	3	68	70	Skin	Basal cell carcinoma	Present	Chondrocytes; endothelial cells; dendritic cells; nerve bundles in skin. Interfollicular/paracortical lymph node parenchyma (T‐cell rich areas of node)	Coincident	12 months
		5	55		Lipoma and lymph node	No malignancy	Present		144 months	168 months
28	sALS	2	74	76	Lymph node	Unsatisfactory	Absent			
		3	74		Lymph node	No malignancy	Absent			
		3	70		Pleura	No malignancy	Absent			
29	*C9orf72*	1	55	66	Skin	Squamous cell carcinoma *in situ*	Absent			
		1	57		Skin	Hyperkeratosis	Absent			
30	sALS	2	67	73	Skin	Pigmented nevus	Absent			
		12	61		Uterus	Adenocarcinoma	Absent			
31	sALS	2	75	78	Gall Bladder	Gallstone disease	Present	Myenteric plexus and lamina propria	12 months	24 months
33	sALS	1	83	84	Skin	Squamous cell carcinoma	Absent			
40	sALS	2	71	74	Skin	Actinic keratosis	Absent			

Clinical demographics and summary of tissue type, diagnosis, and findings from pTDP‐43 staining for the tissue cohort comprising the 13 ALS patients with examinable material.

### Immunohistochemistry and immunofluorescence

Sections were dewaxed using successive xylene washes followed by alcohol hydration and treatment with picric acid for removal of formalin pigment. For pTDP‐43 and CD68 staining, antigen retrieval was carried out in citric acid buffer (pH 6) in a pressure cooker for 30 min, after which immunostaining was performed using the Novolink Polymer detection system (Leica Biosystems, Newcastle, UK) with phospho(409–410)‐TDP43 antibody (CAC‐TIP‐PTD‐M01, 1 in 4000, 2B Scientific, Bicester, UK), or anti‐CD68 (M081401‐2, 1 in 100, Agilent, Cheadle, UK). Phospho‐Tau (Ser202, Thr205) monoclonal antibody (AT8 MN1020, 1 in 2000, ThermoFisher) was used without antigen retrieval. Slides were counterstained with haematoxylin and mounted using DPX mountant. For immunofluorescence, mouse anti‐TDP‐43 C‐terminal (12892‐1‐AP, Proteintech, Manchester, UK) was used in addition to the pTDP‐43 antibody. Secondary antibodies were anti‐mouse, Alexa 488 (A21131, Invitrogen, Inchinnan, UK), and anti‐rabbit Cy5 (711‐175‐152, Jackson‐immunochem, Ely, UK). Sections were imaged using an Olympus BX51 with a Nikon Digital‐Sight camera and immunofluorescent images were taken using an Invitrogen EVOS colour fluorescent microscope. Bleach treatment was performed (where warranted) by incubating slides immediately post antigen retrieval in 10% hydrogen peroxide for 12 h overnight. This step was followed by the normal protocol in full, including the additional 3% hydrogen peroxide step used to block endogenous peroxidase activity, following a previously optimised protocol [13]. Sections were imaged using an Olympus BX51 with a Nikon Digital‐Sight camera and whole slide scanned using a NanoZoomer slide scanner (Hamamatsu, Welwyn Garden City, UK).

## Results

### Establishment of a cohort of ALS non‐CNS tissue

**Figure 1:**
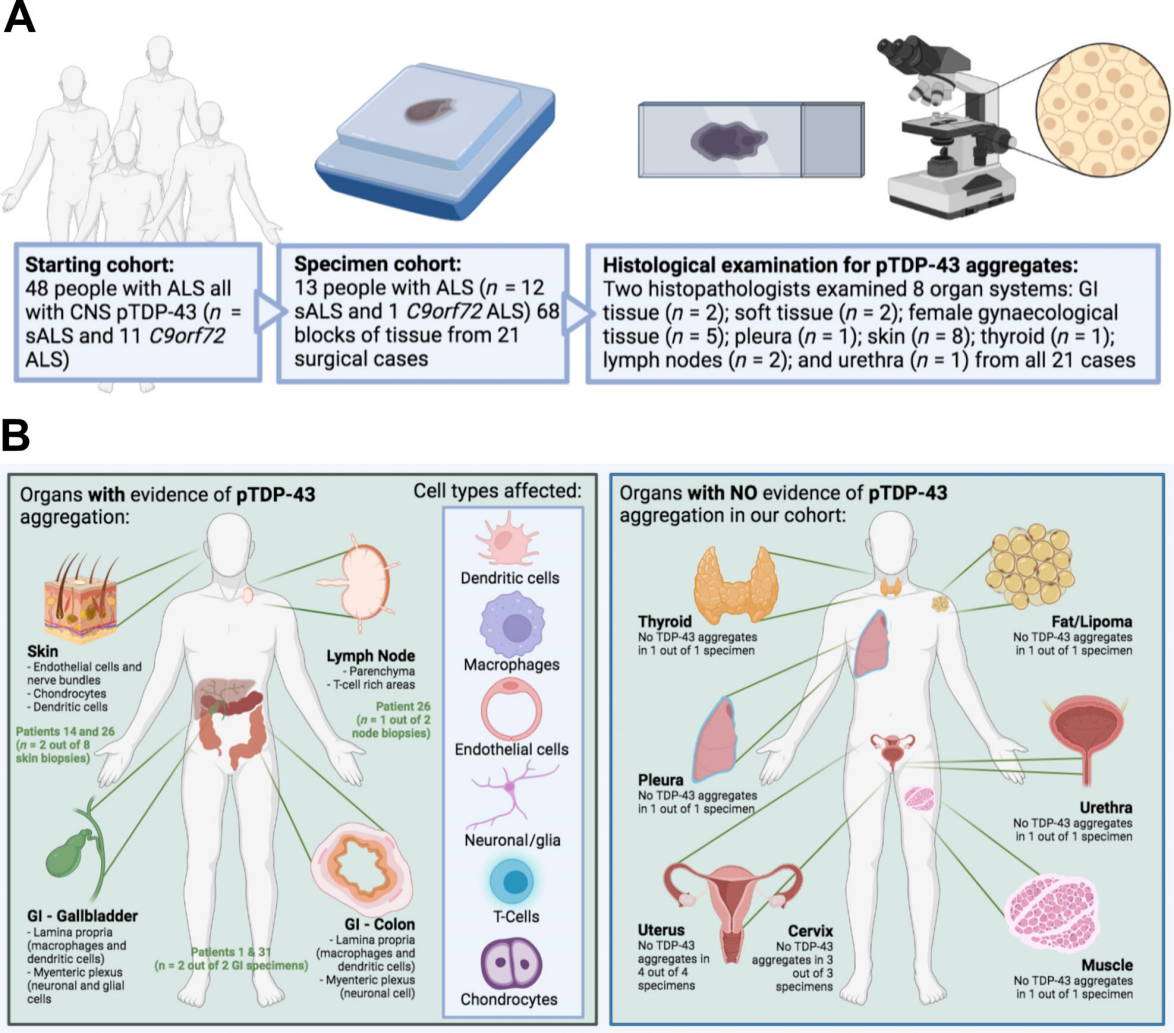
Ante‐mortem tissue cohort comprised of tissue taken from people who went on to develop ALS demonstrates non‐CNS accumulation of pTDP‐43 aggregates prior to symptom onset. Schematic of workflow to identify pTDP‐43 aggregates indicative of non‐CNS manifestations of ALS. Lower panel left: cartoon depicting organs and cell types that had evidence of pTDP‐43 aggregation in ALS patient non‐CNS ante‐mortem tissue. Lower panel right: cartoon depicting organs with no evidence of pTDP‐43 aggregation in ALS patient non‐CNS ante‐mortem tissue.

We have previously evaluated pTDP‐43 staining within the CNS of 48 patients (37 sALS patients and 11 patients with a *C9orf72* hexanucleotide repeat expansion). All 48 patients had pathological pTDP‐43 inclusions in their CNS tissue and a diagnosis of ALS, and their CNS post‐mortem tissue has been evaluated previously in our prior studies [14, 15]. Within this group of 48 individuals with a diagnosis of ALS, 9 had mild cognitive involvement (ALSci; measured by the Edinburgh Cognitive ALS Screening [ECAS] tool) and 1 had a behavioural deficit (ALSbi), but none met criteria for a diagnosis of frontotemporal dementia, and 32 patients had not been evaluated using the ECAS during life. All cases included in the cohort had CNS pTDP‐43 pathology in motor brain regions and in some cases also in non‐motor brain regions [14], and we wanted to establish whether they also had pTDP‐43 pathology within non‐CNS tissue. As such, we requested all non‐CNS tissue from the NHS Lothian BioResource that provides controlled access to all tissue taken during life (i.e. diagnostic and surgical) from all patients under the care of NHS Lothian and Fife, for approved research use (Figure 1). Of the 48 patients that we requested tissue for, 13 had surgical specimens from non‐CNS sites and sufficient tissue for evaluation: 12 patients with sALS and 1 patient with a *C9orf72* hexanucleotide repeat expansion. The final cohort consisted of tissue from 22 surgical cases (some patients having more than one sample over their lifetime), representing eight organ systems: GI tissue (*n* = 2), soft tissue (e.g. lipoma/muscle; *n* = 2), female gynaecological tissue (*n* = 5), pleura (*n* = 1), skin (*n* = 8), thyroid (*n* = 1), lymph nodes (*n* = 2), and urethra (*n* = 1) (Table 1 and Figure 1). Interestingly, pTDP‐43 aggregates were only identified in GI tissue (two of two cases), lymph nodes (one of three cases), and skin (two of eight cases). For those with aggregates, the median time to symptom onset and diagnosis from tissue sampling was 12 months (range = 0–144 months) and 24 months (range = 2–168 months), respectively (Figure 1). Both GI specimens evaluated were taken 2 months and 2 years prior to diagnosis, with the gallbladder specimen showing pTDP‐43 aggregates 1 year prior to the onset of their motor symptoms. Notably, one sample from patient 26 had pTDP‐43 aggregates present in a lymph node 14 years prior to diagnosis with ALS. The remaining tissues showed no evidence of pTDP‐43 pathology other than within blood vessel endothelial cells in 6 of 22 specimens (Table 1).

### Presence of pathological pTDP‐43 aggregates in GI tract of ALS patients prior to motor symptom onset highlights potential for use as early biomarker

**Figure 2:**
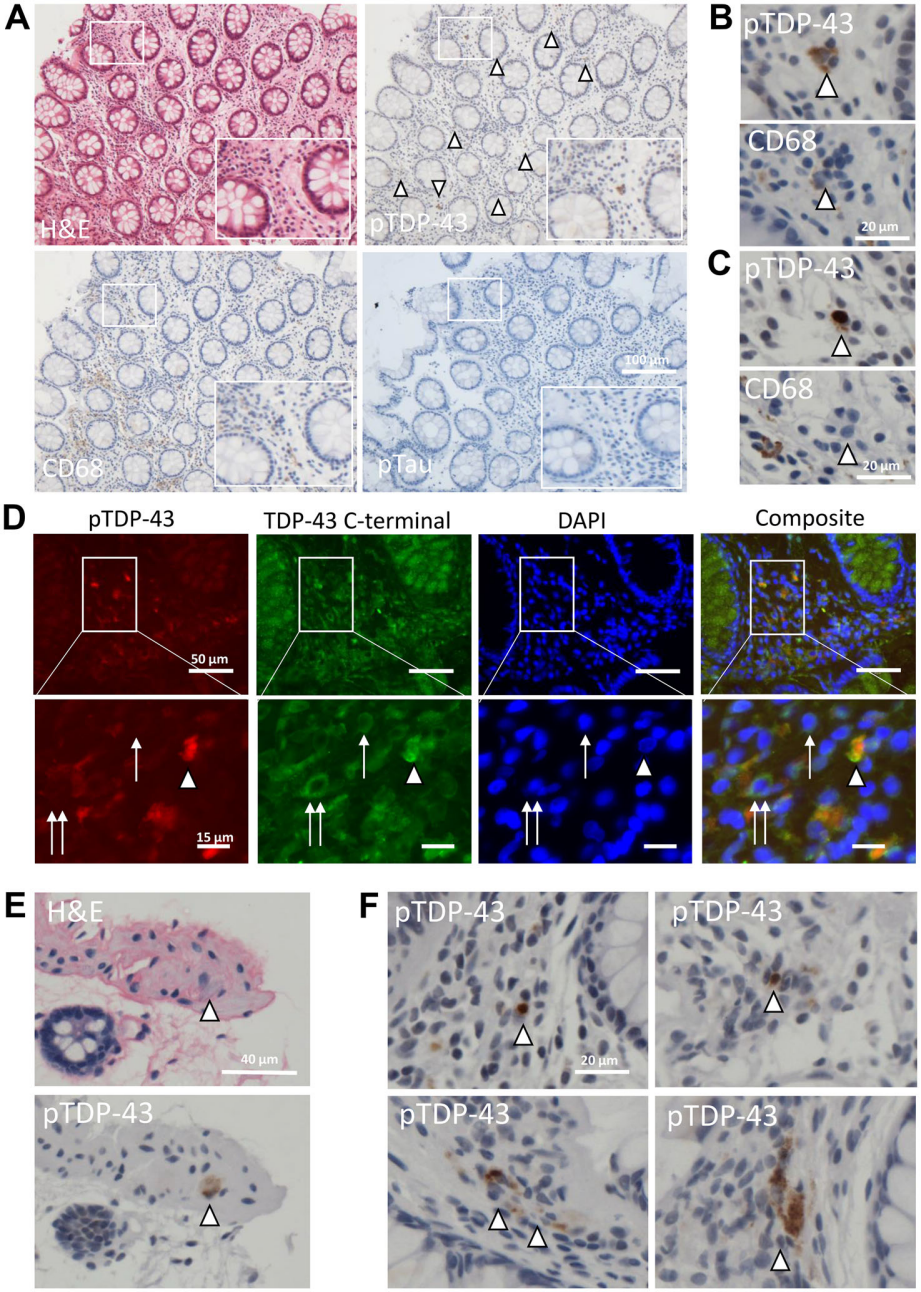
pTDP‐43 aggregates are present in lamina propria and nerve bundles of colonic tissue and demonstrate diverse aggregate morphologies. (A) Photomicrographs of colonic biopsy (patient 1). Top left – H&E; top right – pTDP‐43 (multiple lamina propria aggregates highlighted by white arrow heads); bottom left – CD68 (highlighting activated macrophages); bottom right – pTau (negative). Scale bar = 50 μm, images taken at ×20 magnification. (B) ×40 magnification photomicrograph highlighting, in serial sections, macrophage staining in the same area as the TDP‐43 aggregate staining (i.e. pTDP‐43 present within macrophages, implying active aggregate clearance). (C) ×40 magnification photomicrograph highlighting, in serial sections, no evidence of macrophage staining in the same area as the cell, highlighted with a white arrowhead, containing a dense perinuclear single pTDP‐43 aggregate within a dendritic cell (implying that pTDP‐43 aggregates are also in cell types that are not clearing aggregates, i.e. non‐macrophage cells). (D) Fluorescent micrographs demonstrating pTDP‐43 (red), C‐terminally detected TDP‐43 (green), and DAPI‐stained nuclei in the same GI biopsy as imaged in (A). Arrowhead shows co‐incident staining of mature TDP‐43 aggregates, single arrow shows normal nuclear TDP‐43 staining in cells unaffected by TDP‐43 aggregation, and the double arrow shows early TDP‐43 pathology exemplified by loss of normal nuclear localisation of TDP‐43 into a cytoplasmic location. These images, by using two antibodies directed at two distinct epitopes of TDP‐43 demonstrate TDP‐43 pathology at multiple know stages of the aggregation process, mirroring the temporal aggregation events seen in the CNS at post‐mortem. (E) H&E (top) and pTDP‐43 immunohistochemistry (bottom) images demonstrating pTDP‐43 aggregation in a large neuronal cell within a nerve bundle. (F) Examples of pTDP‐43 aggregate morphology ranging from single, dense perinuclear aggregates (top images) and dispersed cytoplasmic dot‐like aggregates throughout the cytoplasm (bottom images).

Patient 1 (Table 1) had an investigative colonoscopy around the time of ALS symptom onset, 2 months prior to their ALS diagnosis, and the examination revealed a small sessile polyp; three random colonic biopsies were also taken at the time of biopsy. The sessile polyp was a benign, non‐dysplastic hyperplastic polyp and the three random colonic biopsies were reported as normal with no evidence of infection or microscopic colitis and no evidence of dysplasia or malignancy. Both the polyp and the colonic biopsies showed evidence of pTDP‐43 aggregation within the lamina propria (the mucosal connective tissue deep to the surface enterocytes; Figure 2A) with examples of pTDP‐43 aggregates in CD68‐positive (i.e. activated) macrophages (Figure 2B) and in non‐CD68‐positive dendritic cells (Figure 2C). We also probed the mucosal GI biopsy with an alternative TDP‐43 antibody to an epitope in the C‐terminal region. This antibody detects all TDP‐43, not just the pathogenic TDP‐43 aggregates, which are detected by the pTDP‐43 antibody (Figure 2D). Using this antibody in combination with the pTDP‐43 antibody, we see co‐staining of mature cytoplasmic aggregates in the cells affected by cytoplasmic aggregation (red and green co‐incident staining marked by the arrowhead; Figure 2D). However, we also see normal TDP‐43 localisation in the nuclei of unaffected cells (single arrow showing green staining only) and the initial stages of TDP‐43 aggregation, characterised by complete loss of TDP‐43 nuclear staining and redistribution to the cytoplasm (double arrow showing green staining only), a feature known to precede TDP‐43 aggregation in CNS tissues. This range of TDP‐43 aggregation events mirrors CNS tissue at post‐mortem in these cases. No pTDP‐43 immunoreactivity was noted in non‐ALS colon tissue (*n* = 3; supplementary material, Figure S1).

pTDP‐43 aggregates were also visualised within neuronal cells in the nerve bundle sampled in one of the colonic biopsies (Figure 2E). The pTDP‐43 aggregates have multiple morphologies, including single dense perinuclear amorphous cytoplasmic aggregates (Figure 2F; top panel) and scattered dispersed small dot‐like cytoplasmic staining (Figure 2F; bottom panel), similar to that seen in the CNS. The aggregation in these specimens was specific for TDP‐43 proteinopathy; no pTau aggregates were observed (Figure 2A).

**Figure 3:**
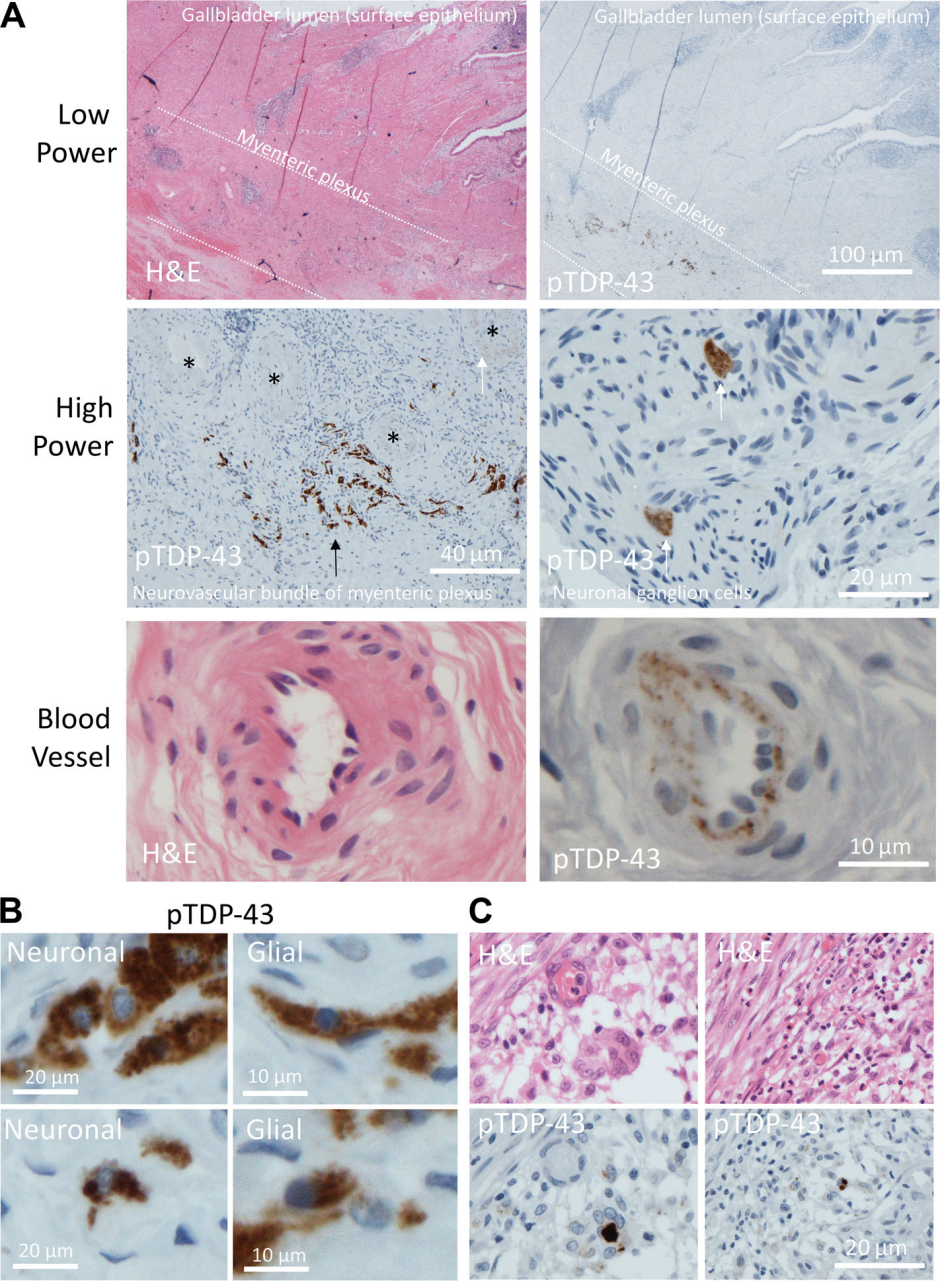
pTDP‐43 aggregates identified in lamina propria and myenteric plexus of gallbladder mucosa. (A) Low power photomicrographs of gallbladder wall (patient 31), top left – H&E illustrating location of epithelial‐lined mucosal surface and myenteric plexus. Top right – pTDP‐43 staining performed on the serial section showing extensive pTDP‐43 aggregation within the myenteric plexus. Middle panel (left) high power view of myenteric plexus, with aggregates in the neuronal cells (black arrow) and endothelial cells (white arrow) of the plexus. Black asterisks show blood vessels cut in transverse section. Middle panel (right) two neuronal cells demonstrating cytoplasmic pTDP‐43 aggregation within a nerve bundle in the wall of the gallbladder. Lower panel (left) H&E and (right) pTDP‐43 staining demonstrating blood vessel endothelial cells cytoplasmic aggregates. (B) Example photomicrographs demonstrating aggregates in cells with neuronal morphology (left) and glial morphology (right). (C) Left and right demonstrate H&E (top) and pTDP‐43 performed on the serial section (bottom), demonstrating extensive lamina propria and mucosal aggregates within dendritic cells and macrophages.

Patient 31 (Table 1) had a cholecystectomy 12 months prior to ALS symptom onset and 24 months prior to their ALS diagnosis, and the gallbladder was reported as showing chronic cholecystitis secondary to gallstones. pTDP‐43 staining revealed extensive aggregate formation in neuronal and glial cells within the myenteric plexus (the primary neurovascular bundle of the submucosal tissue) of the gallbladder (Figure 3A,B). Aggregates were also noted within endothelial cells of small‐ to medium‐sized blood vessels within the myenteric plexus (Figure 3A). There was also evidence of extensive pTDP‐43 aggregate formation within dendritic cells and macrophages in the lamina propria, as seen in the colonic mucosa (Figure 3C).

### Pathological pTDP‐43 aggregates are identified in lymph node parenchyma and specialised cells of the skin

**Figure 4:**
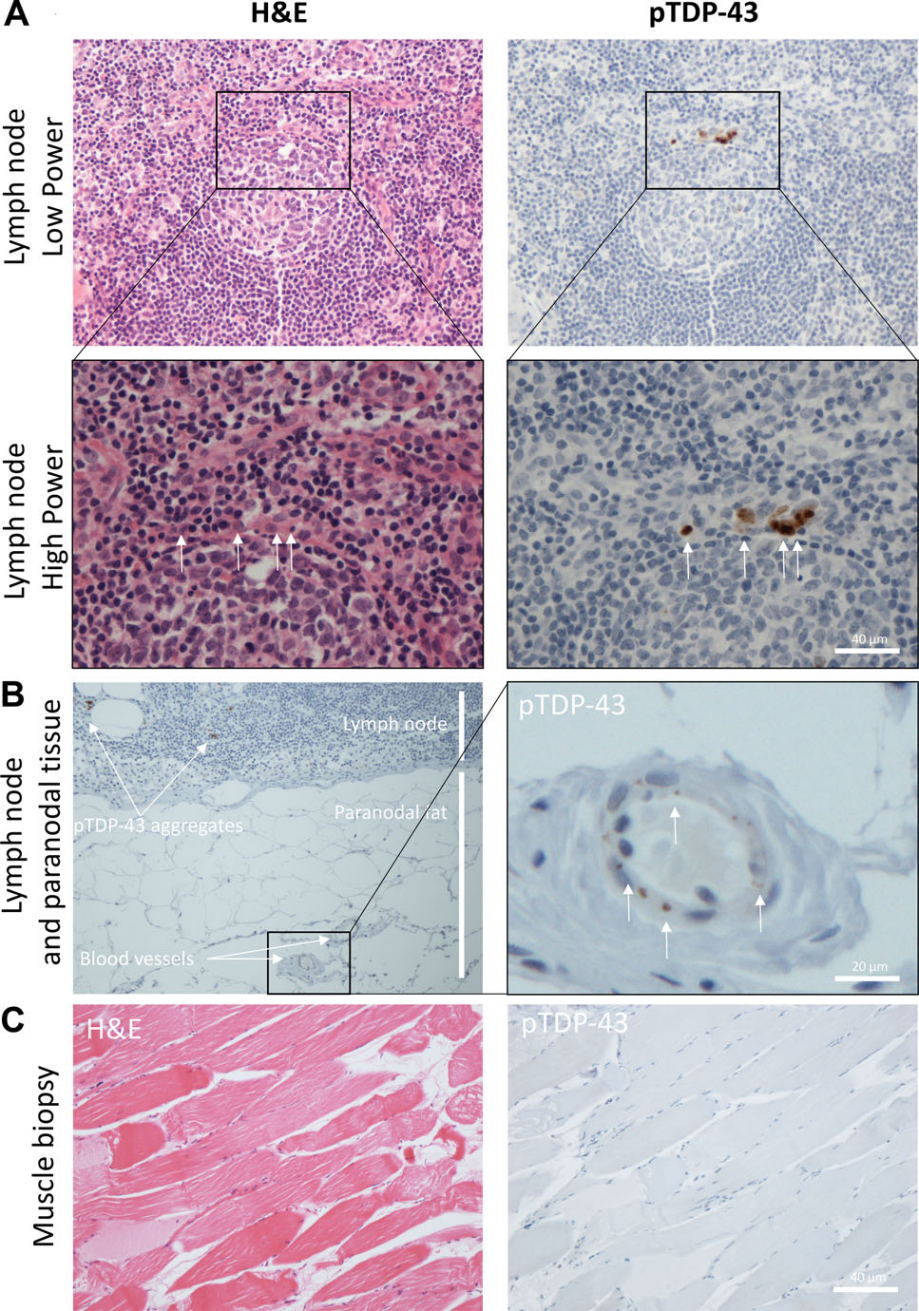
pTDP‐43 aggregates identified in lymph node parenchyma, endothelial cells, and chondrocytes long before symptom onset of ALS. (A) H&E (left) and pTDP‐43 (right), low power (top) and high power (bottom) images of an active lymph node germinal centre with evidence of cells containing pTDP‐43 aggregates (white arrows) within the mantle zone rim of the lymphoid follicle. (B) Low power (left) and high power (right) images of the paranodal tissue demonstrating blood vessel pTDP‐43 aggregation (white arrows) in adjacent feeder vessels. (C) H&E (left) and pTDP‐43 (right) images showing no evidence of pTDP‐43 aggregates in a muscle biopsy taken at point of diagnosis from patient 1.

**Figure 5:**
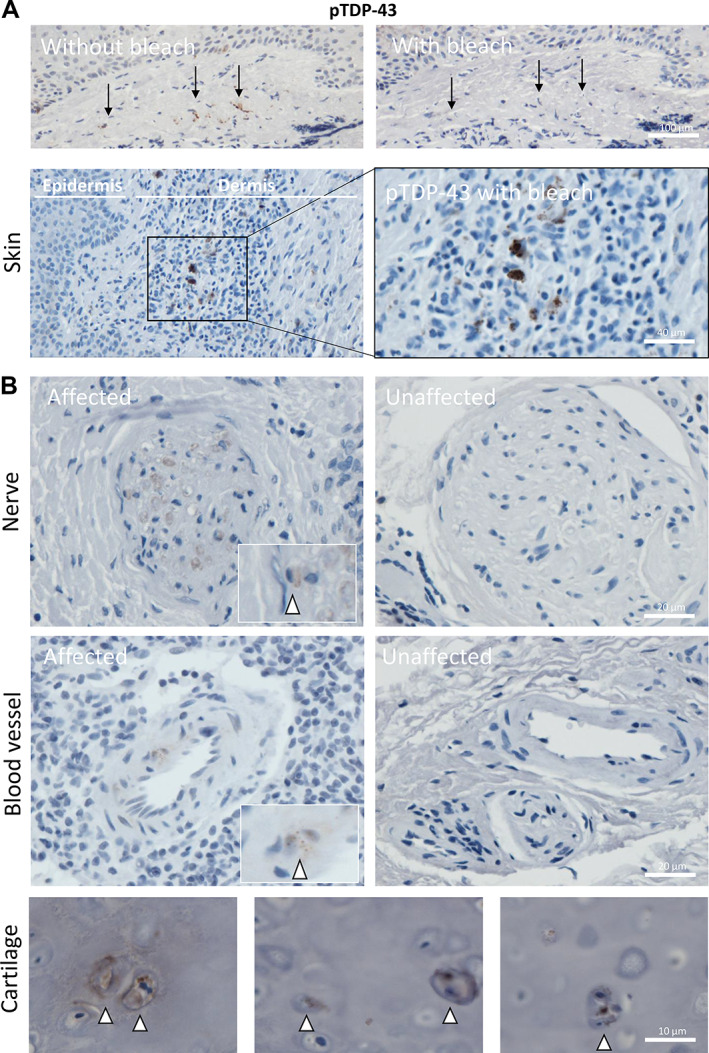
pTDP‐43 aggregates identified in nerve bundles, endothelial cells, and superficial dermis of skin. (A) Images demonstrating pTDP‐43 staining of skin following removal of brown pigmentation (from melanocytes and/or pigment drop out as a reactive feature) from the dermis and epidermis (above) and pTDP‐43 positive aggregates within the superficial dermis (below). All are taken from patient 26. (B) Example of pTDP‐43 present within peripheral nerves (top left; patient 14) and an example of an unaffected nerve bundle (top right; patient 29) and within endothelial cells of the dermal blood vessels (bottom left; patient 14) and an unaffected blood vessel for comparison (bottom right; patient 29). (C) Three example images taken from patient 26 demonstrating pTDP‐43 aggregates within the chondrocytes of the ear cartilage sampled as part of an excision specimen for a basal cell carcinoma.

Patient 26 had several specimens taken several years apart, all prior to their diagnosis of ALS. The first specimen of note was a probable lipoma taken along with a supraclavicular lymph node 14 years prior to their ALS diagnosis and 12 years before they had ALS‐associated motor symptoms. The specimen, at the time of sampling, was reported as a lipoma and normal lymph node with no evidence of malignancy. Our examination revealed the lymph node to contain cells that stained positively for pTDP‐43 (Figure 4). These cells appeared within the interfollicular/paracortical lymph node parenchyma, predominantly in T‐cell‐rich and antigen‐presenting regions (Figure 4A). pTDP‐43 aggregates were also found within blood vessels in the paranodal tissue (Figure 4B). Of note, no pTDP‐43 aggregates were found in the muscle biopsy sampled at time of symptom onset, 2 months prior to their ALS diagnosis (Figure 4C). The next specimen of note was a skin sample taken from the ear around the time of their symptom onset, 12 months prior to their ALS diagnosis, with a clinical suspicion of basal cell carcinoma and was reported as a completely excised basal cell carcinoma. The specimen showed evidence of pTDP‐43 aggregates within several cell types and regions: (1) the superficial dermis of the skin (Figure 5A); (2) within endothelial cells of small‐ to medium‐sized blood vessels; (3) within Schwann cells of peripheral nerve bundles; and (4) within chondrocytes (cartilage‐producing mesenchymal cells which may have a shared lineage with neurons in the neural crest) (Figure 5B). The superficial dermis, neurons, and endothelial cells were also seen to be involved by pathological pTDP‐43 aggregates in tissues sampled from other cases (see Table 1 and Figure 1 for a summary of affected tissues).

## Discussion

Here we show evidence of pTDP‐43 aggregation in human GI tissue taken as part of routine clinical practice during life from ALS patients prior to diagnosis of their motor symptoms. This finding is in line with gut biopsies taken from patients who went on to develop PD, where alpha‐synuclein pathology can be seen many years prior to diagnosis [5, 16]. Patients with neurodegenerative diseases frequently report GI symptoms prior to their neurological diagnosis [1]. Our data, as well as that published for alpha‐synuclein in PD patients, support the hypothesis that GI pathology is present before CNS symptoms occur. Indeed, these findings are not only useful as potential targets for early biomarkers and to improve stratification for clinical trials, but they could also be used to develop tissue biopsy approaches to understand target engagement in trials aimed at reducing TDP‐43 or alpha‐synuclein aggregation burden. A GI biopsy or even a stool sample would circumvent the issue of not being able to biopsy CNS tissues.

This raises the question of how pathology arising in the gut might reach the CNS. In our data, and in that published for alpha‐synuclein, aggregates are seen in both the lamina propria and the myenteric plexus. Antigens, including soluble molecules of comparable size to TDP‐43 and alpha‐synuclein, have been shown by two‐photon imaging to enter the lumen‐facing surface of intestinal goblet cells and be transported to be presented to dendritic cells within the lamina propria [17, 18]. This is referred to as goblet‐cell‐associated antigen passage and is restricted to small molecules up to 70 kDa in size. M cells are gut resident surface epithelial cells thought to be responsible for this selective endocytosis of antigens, directly transporting them to tissue‐resident macrophages and local lymphocytes, which then migrate to lymph nodes where an immune response can be initiated [17, 18], as seen in the gallbladder tissue in our cohort. Alternatively, a physical connection to the CNS has been posited in the animal model literature; that we also detect pTDP‐43 aggregates within endothelial cells and in lymph nodes raises the possibility of dissemination through a non‐physical connection. Furthermore, that we also see pTDP‐43 aggregates in chondrocytes of the ear cartilage raise the further possibility of meta‐synchronous aggregation occurring in a susceptible individual in certain cell types (i.e. occurring separately in a non‐linked fashion due to similar environmental stimuli). Of note, chondrocytes are not unlike motor neurons, as they are both long‐lived post‐mitotic cells derived from the neural crest. Therefore, a shared susceptibility to meta‐synchronous protein aggregation is possible.

Whilst the presence of pTDP‐43 aggregates within gut tissues in our cohort raises the possibility of a similar mechanism of pathological spread to that seen in other neurodegenerative diseases (as detailed in the early preclinical studies outlined above), we also see pTDP‐43 in other tissues including skin, peripheral nerves, and blood vessels as well as lymph nodes that would not typically drain lymph fluid from the GI tract. Other groups have identified pTDP‐43 aggregates within peripheral nerves, with an identical staining pattern to that seen in our cohort [7]. Several studies have also evaluated TDP‐43 pathology in muscle biopsies [8, 9]. One study showed TDP‐43 pathology in 19 of 57 ALS cases [9] but a further study showed no evidence of TDP‐43 pathology in the 3 cases that they evaluated [8]. Our cohort only included one muscle specimen; however, we detected no evidence of pTDP‐43 pathology in this case. We detected pTDP‐43 aggregates in two of eight skin biopsies. Indeed, a previous study demonstrated an overall increase in immunoreactivity of full‐length TDP‐43 in the epidermis of ALS patients (*n* = 15) compared to controls (*n* = 15) [19]. Although they did not identify aggregation *per se*, increased levels of highly aggregation‐prone proteins like TDP‐43 may be indicative of pathological TDP‐43 turnover that could predispose these individuals to TDP‐43 aggregation. Indeed, these data from other cohorts perhaps indicate that more than just the gut‐brain‐axis is involved by pTDP‐43 aggregation, supporting the hypothesis of meta‐synchronous aggregation, rather than physical spreading. The mechanistic consequences of the involvement of other tissues and their contribution to ALS pathogenesis clearly require further investigation.

TDP‐43 pathology has been noted in post‐mortem cerebral blood vessels previously [20, 21]. However, the presence of pTDP‐43 aggregates within peripheral blood vessels in 6 of 13 presymptomatic cases evaluated in our cohort raises the possibility that TDP‐43 pathology could be identified prior to neurological dysfunction in ALS, holding promise as an early detector of pathology. Indeed, endothelial cell involvement by pTDP‐43 aggregation could directly result in misfolded forms of TDP‐43 in circulating blood, which could be detected in blood samples taken from these individuals prior to symptom onset. Indeed, all cases with endothelial involvement in our cohort had pathology detectable in tissues prior to ALS diagnosis. Furthermore, we have shown previously by systematic review and meta‐analysis that serum‐derived TDP‐43 may show promise as a peripheral biomarker in ALS [22].

Our data imply that peripheral non‐CNS tissues could hold promise as early indicators of ALS, prior to neurological involvement, and that tissue biopsies could also help us to understand how fluid biomarkers, such as blood and stool samples could be utilised through their association with the tissues that they derive from. Early diagnosis in this way would not just improve the chances of successful therapeutic intervention, it would also inevitably lead to a rethinking of neurodegenerative conditions from being end‐stage neurological disorders to systemic disorders with potential for population screening and early intervention long before neurological manifestations occur. Future studies assessing peripheral tissue cohorts such as these using western blotting might uncover the presence of C‐terminal fragmentation that is also a distinguishing, albeit heterogenous, feature of pathological aggregates in ALS [23]. Furthermore, this could provide further conformational information such as tissue and even cell‐type specific species of TDP‐43, as has been done in CNS tissue and which could further inform future biomarker studies [24].

### Study limitations

Our study was based on a cohort of 48 patients, only 13 of which had surgical specimens that were sufficient for histological assessment, 12 of which were sALS cases. Therefore, the conclusions of this study are subject to the caveats of small sample size and are biased towards sALS. For example, with tissues that we have seen no evidence of aggregation in our cohort, we cannot rule out that they are not involved by pTDP‐43 aggregation in a larger cohort, we can only state that they are not involved in our cohort. Furthermore, tissue cohorts such as these are necessarily subject to variations in fixation and ischaemic times during excision and post‐excision processing, all of which may affect our ability to detect proteins, particularly phosphorylated proteins, resulting in the possibility of false negatives. It is therefore important to include all relevant information and detailed methodological reporting to facilitate future meta‐analysis where more cases and cohorts can be evaluated in pooled datasets, as has been done for alpha‐synuclein [25]. For this reason, we have included detailed clinical information and histological images for future analysis. Another relevant caveat of this work is that we selected only ALS patients known to have CNS pTDP‐43 pathology. This is therefore a selected cohort and does not allow us to understand population distribution of these pathologies in unaffected individuals. A future study evaluating larger numbers of unselected individuals is now warranted to understand the population dynamics of this pathology and provide more information regarding the feasibility of population screening and the establishment of larger cohorts for molecular studies. However, a strength of the approach we have taken is that surgical biopsies deposited in Biorepositories allow us to gain a better understanding of the temporal nature of the development of ALS and the involvement of non‐CNS tissues in this process.

## Author contributions statement

SBP, MJA, MH, FMW and JMG contributed to conceptualisation, project administration, resources, supervision, validation, and visualisation. All authors contributed equally to data curation and formal analysis. JMG contributed to funding acquisition. SBP is a data pathologist and contributed to study design, cohort identification, and data blinding necessary for the use of NHS BioRepository tissues. SBP, JO'S, MJA, MH, FMW and JMG contributed to investigation and methodology. SBP, FMW and JMG wrote the first draft of the manuscript prior to other author input. JO'S, JP, OK, OR, KN and NH were involved with tissue/data handling and/or performed all tissue staining, imaging, and whole slide scanning. All authors contributed to manuscript reviewing and editing.

## Supporting information


**Figure S1.** pTDP‐43 aggregates are not identified in colon biopsies from non‐ALS individuals

## Data Availability

All clinical and demographic data have been shared in an anonymised format as per ethical restrictions of tissue use from the NHS BioResource. All slides processed in this study have been whole slide scanned and can be requested from the first or senior author and will be transferred as .ndpi files which can be visualised using freely available software. All other data are included in their entirety within the manuscript.
