# Supplementary material for: pTDP‐43 aggregates accumulate in non‐central nervous system tissues prior to symptom onset in amyotrophic lateral sclerosis: a case series linking archival surgical biopsies with clinical phenotypic data
Source: J Pathol Clin Res. 2022 Oct 13;9(1):44–55. doi: 10.1002/cjp2.297 (PMC9732680; doi:10.1002/cjp2.297)
Supplement: Supplementary file 1 — Figure S1. pTDP‐43 aggregates are not identified in colon biopsies from non‐ALS individuals [file CJP2-9-44-s001.pdf]

pTDP-43 aggregates accumulate in non-central nervous system tissues prior to symptom onset in amyotrophic lateral sclerosis: a case series linking archival surgical biopsies with clinical phenotypic data

SB Pattle, J O'Shaughnessy *et al. J Pathol Clin Res* https://doi.org/10.1002/cjp2.297

Supplementary Figure S1

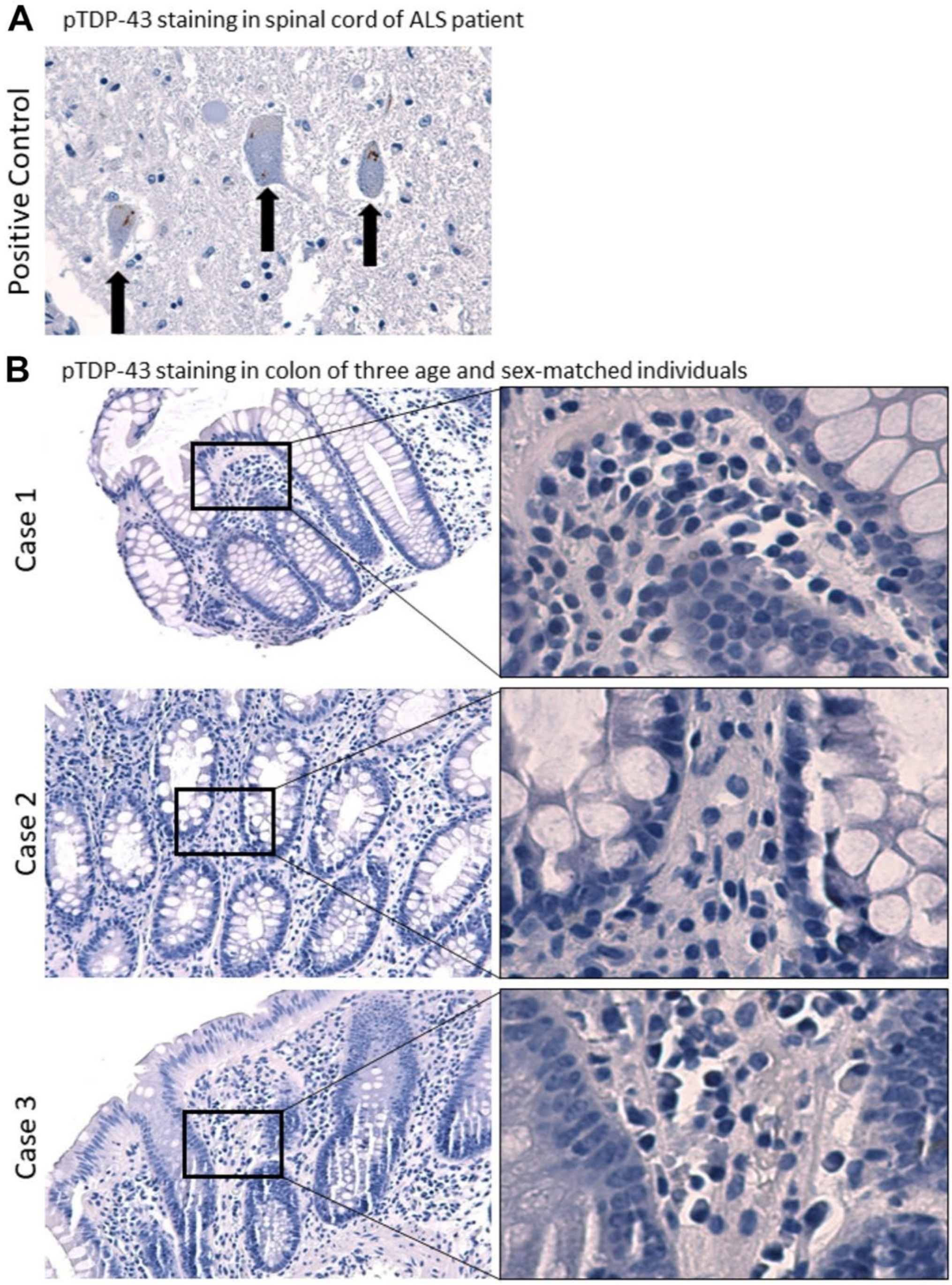

Figure S1. pTDP-43 aggregates not identified in colon biopsies from non-ALS individuals. (A) Photomicrograph demonstrating a positive control showing pTDP-43 aggregates in the spinal cord of an individual with ALS (black arrows). (**B)** Photomicrographs demonstrating no evidence of pTDP-43 immunoreactivity in population individuals not selected from the ALS cohort (n=3 individuals).
